# Physiological and Biochemical Mechanisms Behind Enhanced Salinity Tolerance in *Limonium irtaense* Seedlings Following Recovery from Salt Stress

**DOI:** 10.3390/plants15030451

**Published:** 2026-02-01

**Authors:** Diana-Maria Mircea, Adrián Sapiña-Solano, Eloy Molina, P. Pablo Ferrer-Gallego, Antonio Lidón, Jaime Prohens, Ricardo Mir, Oscar Vicente, Monica Boscaiu

**Affiliations:** 1 Mediterranean Agroforestry Institute (IAM), Universitat Politècnica de València, Camino de Vera s/n, 46022 Valencia, Spain; dmircea@doctor.upv.es (D.-M.M.); mobosnea@eaf.upv.es (M.B.); 2 Institute for the Conservation and Improvement of Valencian Agrodiversity (COMAV), Universitat Politècnica de València, Camino de Vera s/n, 46022 Valencia, Spain; adsaso@doctor.upv.es (A.S.-S.); jprohens@btc.upv.es (J.P.); rimimo@upvnet.upv.es (R.M.); 3 Department of Agroforest Ecosystems, Universitat Politècnica de València, Camino de Vera s/n, 46022 Valencia, Spain; emolcar@epsg.upv.es; 4 Wildlife and Natura 2000 Service, Generalitat Valenciana, Avda Comarques del País Valencia, 114, Quart de Poblet, 46930 Valencia, Spain; pferrergallego@gmail.com; 5 Research Institute of Water and Environmental Engineering (IIAMA), Universitat Politècnica de València, Camino de Vera s/n, 46022 Valencia, Spain; alidon@qim.upv.es

**Keywords:** abiotic stress, salt stress biomarkers, endemism, seed germination, vegetative growth

## Abstract

*Limonium irtaense* is an endangered halophyte endemic to coastal Castellón (Spain). This study aimed to support its conservation by assessing the effects of salinity on seed germination and seedling performance, as well as plants’ physiological and biochemical responses to salt stress during early vegetative growth. Seed germination was tested in the presence of 0 to 300 mM NaCl, followed by recovery assays for non-germinated seeds. Seedlings were grown under three salinity levels, by irrigation with water (control), 300 mM NaCl or 600 mM NaCl. Growth parameters, photosynthetic pigments, osmolytes, ion contents, oxidative stress markers and antioxidant compounds were determined in plants derived from the initial germination tests and the recovery of germination assays and subjected to the different salt treatments. Germination was highest in distilled water and declined with increasing salinity; however, salt-inhibited seeds germinated rapidly and efficiently in the recovery assays. Seedlings from salt-primed seeds showed higher survival rates and biomass than those from control germination tests. Salt treatments significantly reduced growth, with plants derived from salt-treated seeds generally showing higher tolerance, probably because of enhanced proline accumulation, more efficient transport and sequestration of toxic ions in leaf vacuoles, and potassium retention. These findings provide insights into *L. irtaense* adaptation mechanisms and support using salt-priming to improve conservation and translocation efforts for this endangered species.

## 1. Introduction

The Iberian Peninsula harbours almost 6500 species and subspecies of vascular plants, out of which more than one-third are endemic to the region [1]. The Valencian Community, located in eastern Spain, is distinguished by its rich floristic diversity, which is attributed to its unique geographical, climatic, and geological conditions. This region harbours a range of specialised habitats that sustain a high number of endemic plant species, including many confined to vulnerable environments prone to anthropogenic disturbances [2,3]. Conservation of this rich biodiversity is crucial for maintaining ecosystem balance and promoting human well-being. However, in recent decades, intense land use driven by economic development, particularly coastal tourism, has led to a significant decline in plant species, exacerbating the risk of extinction for rare and endemic taxa. In response, conservation efforts have been strengthened, with a particular focus on monitoring endangered flora and developing conservation strategies, such as seed banking and translocation [4,5].

One such species, *Limonium irtaense* Ferrer et al., was first identified in 2012 and described in 2015 from a single population located in the Sierra de Irta, near Peñíscola, Castellón, Valencian Community, Spain [6]. Initial estimates indicated that fewer than 20 individuals remained in the world’s only known natural population. Since then, conservation efforts, including translocations and propagation in nurseries, have helped increase the population of this endemic plant [7]. However, significant challenges remain, particularly considering environmental stressors such as salinity, which is expected to increase as one of the effects of climate change and could affect the species’ viability.

Seed germination and seedling establishment are critical, high-risk stages in a plant’s life cycle, shaping population dynamics and recruitment [8]. In endangered species like *L. irtaense*, successful recruitment is vital for population persistence [9]. Many rare species decline due to constraints in diaspore development, dispersal, or germination. Recruitment depends on seed supply, genetic traits, viability, adaptability, and site conditions [10], with habitat attributes strongly affecting emergence [11]. Failures often result from unsuccessful germination, seedling mortality, or juvenile loss due to harsh environments [12,13]. Identifying traits influencing these stages is essential for effective conservation planning.

Seed germination is a developmental phase that is especially vulnerable to salinity. Even in halophytes, germination is usually optimal without salt and inhibited at levels far below those tolerated by adults [14,15]. Responses vary between species and reflect ecological adaptations and habitat salinity patterns. Many halophyte seeds maintain viability under low water potential, remaining dormant under high salinity conditions but germinating rapidly when returned to fresh water. This distinguishes them from salt-sensitive species, whose seeds lose viability after prolonged exposure to salts. Salt-induced dormancy, common in halophytes, prevents germination during periods of high salinity, such as seawater flooding, thereby protecting seedlings [16]. This strategy maintains a persistent seed bank that germinates when salinity falls within tolerance limits, offering a selective advantage in fluctuating coastal environments [17].

Unlike glycophytes, which cannot survive or reproduce in saline environments, halophytes can complete their life cycles under such conditions. However, salt stress, like other abiotic stresses, often inhibits growth. While most glycophytes and many halophytes perform best in non-saline conditions, only a few highly tolerant dicotyledonous halophytes show enhanced growth at moderate salinity (50–250 mM NaCl), with reductions at higher levels [14]. Growth decline is largely due to reduced photosynthesis from stomatal closure, limiting CO_2_ uptake [18]. Salt stress combines two stressful components, osmotic stress and ion toxicity. Osmotic stress, shared with drought and temperature extremes, causes dehydration and reduced turgor [19,20]. Ion toxicity from excess Na^+^ and Cl^−^ disrupts enzyme activity, protein synthesis, and nutrient uptake [21]. Sodium further impairs mineral nutrition by competing with K^+^ and Ca^2+^ uptake and contributes to oxidative stress via the increased production of reactive oxygen species (ROS) [22]. Tolerance depends on the vacuolar compartmentalisation of toxic ions to maintain cytoplasmic ionic homeostasis and protect metabolism [19,23], as well as the accumulation of compatible solutes, such as sugars (trehalose, sucrose), polyols (mannitol, glycerol), and amino acid derivatives (proline, glycine betaine). These osmolytes help retain water, stabilise proteins and membranes, and act as antioxidants [24,25].

This is the first study investigating the salinity tolerance of *L. irtaense*, during seed germination and early vegetative growth, to assess the species’ ability to cope with salt stress across these sensitive life stages. Gaining insight into its responses to varying salinity levels, particularly after germination recovery, is key to informing effective conservation strategies. We hypothesised that exposure of *L. irtaense* seeds to salinity during germination could induce a salt-priming effect, such that plants derived from salt-exposed seeds would exhibit enhanced survival, growth performance, and improved salt tolerance when subsequently subjected to saline irrigation, compared with plants originating from seeds germinated under non-saline conditions.

To test this hypothesis and help propose underlying mechanisms, the specific objectives of this study were to: (i) evaluate seed germination under saline conditions and assess the potential for recovery following salt stress; (ii) examine early vegetative growth under different salt concentrations by measuring growth parameters in plants derived from the previous germination trials; (iii) analyse the plant’s responses to salinity, determining the levels of biochemical stress markers (photosynthetic pigments, osmolytes, oxidative stress markers and antioxidant compounds) after the salt treatments; and (iv) assess ion transport regulation by quantifying the concentrations of Na^+^, K^+^, and Cl^−^ in roots and leaves. By integrating these germination, physiological, and biochemical analyses, this study provides a comprehensive assessment of the factors influencing the establishment and stress resilience of *Limonium irtaense*, contributing valuable insights for developing effective conservation and restoration strategies.

## 2. Results

### 2.1. Seed Germination and Recovery Capacity

**Figure 1:**
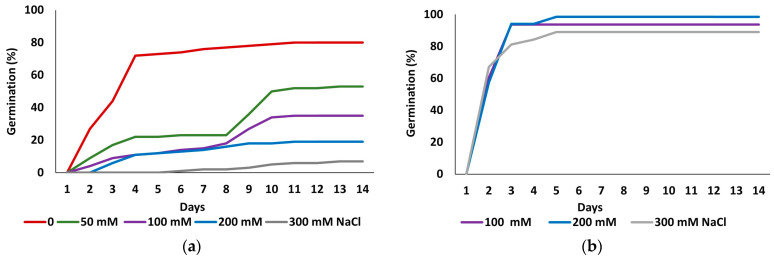
Germination patterns of *Limonium irtaense* seeds observed over 14 days at the indicated salt concentrations (**a**), and germination recovery in distilled water for seeds that failed to germinate at 100, 200 or 300 mM NaCl (**b**).

Figure 1 shows the kinetics of seed germination in the initial germination tests and the recovery experiments. The highest germination percentage was recorded in the control treatment without salt. As the salt concentration increased, germination was delayed, and final germination percentages progressively declined, reaching a minimum at the highest applied concentration of 300 mM NaCl (Figure 1a). In contrast, germination recovery capacity of seeds that did not germinate in the presence of 100, 200 or 300 mM NaCl, was notably high, with values surpassing those observed in the initial control treatment and reaching maximum values within two to three days of incubation (Figure 1b).

Table 1 shows final germination percentages and mean germination times (MGTs) for both germination assays. The statistical analysis of the data revealed significant differences in final germination percentages between all treatments. The control treatment exhibited the highest germination rate (80%), declining progressively in parallel with the increase in salt concentration, down to an average of 7% at 300 mM NaCl (Figure 1a, Table 1). Germination percentages in all recovery treatments, following exposure to 100, 200, and 300 mM NaCl, were significantly higher than in the initial control (*p* ≤ 0.05), reaching values of 93.75%, 98.5%, and 89.06%, respectively, which suggests a beneficial effect of prior salt exposure on germination recovery (Figure 1b, Table 1).

MGT was inversely related to germination percentage, increasing with higher salt concentrations. For instance, seeds subjected to 300 mM NaCl exhibited an average germination time of 9 days, compared to 3.5 days for seeds germinated in water. In contrast, recovery treatments showed enhanced germination and reduced MGT, which averaged approximately 2.4 days, significantly lower than the control, indicating again an overall enhancement in germination performance (Table 1).

### 2.2. Seedling Survival After Transplantation

Table 2 presents the number of seedlings obtained from each germination treatment and the number that survived one month after transplantation into individual pots in the greenhouse. Seedlings derived from the 200 and 300 mM NaCl treatments were not transplanted due to the low number of seeds that germinated under these salinity conditions. Seedling survival rates varied significantly depending on the germination treatment. Notably, only 11.3% of seedlings originating from the control germination treatment survived one month post-transplantation such that only nine seedlings were available for further analyses. In contrast, seedlings from salt stress (50 and 100 mM NaCl) or recovery treatments exhibited markedly higher survival rates. A particularly high survival rate of over 70% was observed in the recovery treatments (R100, R200, R300; Table 2). These results suggest that prior exposure to salt, especially when followed by recovery, may enhance seedling resilience during the early establishment phase.

### 2.3. Early Seedling Growth After Transplantation

Several morphological traits were significantly affected after one month of irrigating the plants with 0 (T1), 300 (T2), or 600 mM NaCl (T3). A two-way ANOVA (Table 3) revealed that the salt treatment significantly affected all analysed growth parameters, except the water content of roots (RWC). In addition, plant origin, defined by the previous germination conditions (O1: 0 mM NaCl, O2: 50 mM NaCl, O3: 100 mM NaCl, O4: R100, O5: R200, O6: R300), also significantly influenced the fresh weight of roots (RFW) and leaves (LFW), as well as leaf water content (LWC). The interaction of the two factors, treatment × origin, was significant for all analysed parameters except LFW (Table 3).

**Figure 2:**
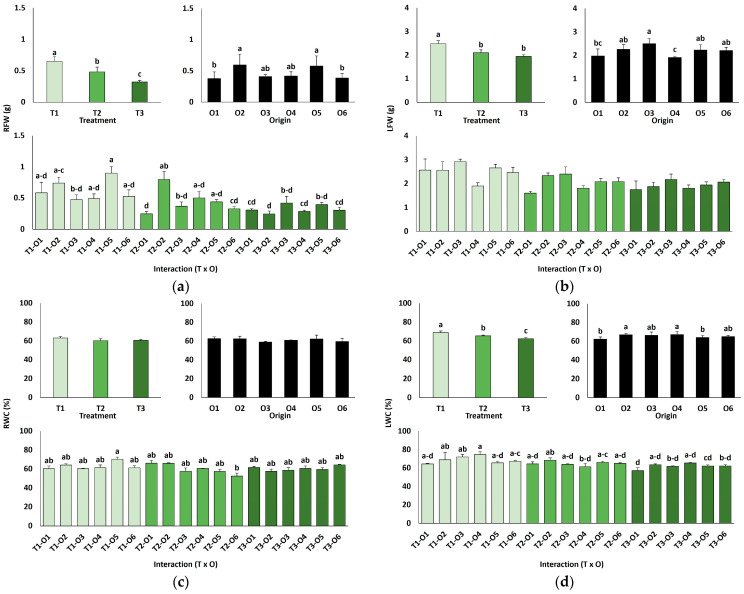
Growth parameters of plants grown from seeds germinated in the indicated conditions (O1: 0 mM NaCl, O2: 50 mM NaCl, O3: 100 mM NaCl, O4: R100, O5: R200, O6: R300; where R100, R200, and R300 refer to germination recovery treatments) after one month of watering with 0 (T1), 300 (T2) or 600 mM (T3) NaCl solutions. Fresh weight of roots (**a**), fresh weight of leaves (**b**), water content of roots (**c**) and water content of leaves (**d**). The values represent means ± SE (*n* = 3 for control treatments, germination without salt; *n* = 5 for seed germination in 100 mM NaCl; *n* = 10 for all other treatments). Different lowercase letters indicate significant post hoc pairwise differences according to the Tukey test at *p* ≤ 0.05, between salt treatments in the greenhouse (treatment), seed germination conditions (origin) and their interaction (T × O).

In line with the ANOVA results, the salt treatments caused a significant inhibition of plant growth, as shown by the concentration-dependent decrease in root (Figure 2a) and leaf (Figure 2b) fresh weight, as well as the reduction in leaf water content (Figure 2d) since salt stress led to partial leaf dehydration. In any case, growth inhibition was neither pronounced nor visually apparent. Plants derived from control seeds germinated directly in water generally showed lower average biomass (root fresh weight, Figure 2a, and leaf fresh weight, Figure 2b) than those grown from seeds germinated directly in the presence of salt or from the recovery treatments, although the differences were small and only in some cases statistically significant (Figure 2).

### 2.4. Biochemical Responses and Ion Accumulation

As shown in Table 4, the salt treatments significantly influenced most biochemical parameters analysed, including photosynthetic pigments contents; ion concentrations in roots and leaves; and proline, hydrogen peroxide, and total phenolic compounds levels. Conversely, plant origin (germination conditions) had a low impact on most of these parameters, with some exceptions, particularly chloride and potassium levels in leaves, or glycine betaine, total phenolic compounds, and total flavonoids contents. However, significant interaction effects between treatment and plant origin were observed for photosynthetic pigments, ion contents in leaves, proline and glycine betaine concentrations, hydrogen peroxide levels, total phenolic compounds, and total flavonoids (Table 4).

**Figure 3:**
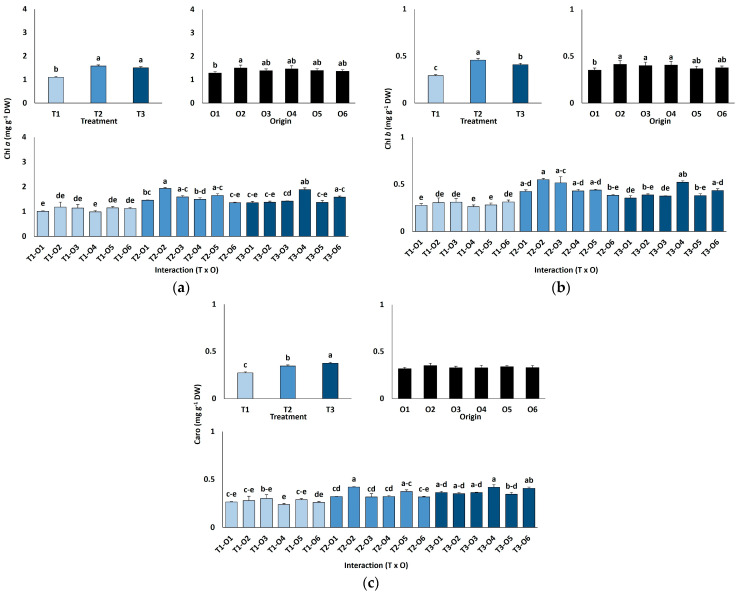
Chlorophyll *a* (**a**), chlorophyll *b* (**b**), and carotenoid (**c**) contents in *Limonium irtaense* plants grown from seeds germinated in the indicated conditions (O1: 0 mM NaCl, O2: 50 mM NaCl, O3: 100 mM NaCl, O4: R100, O5: R200, and O6: R300, where R100, R200, and R300 refer to germination recovery treatments) after one month of watering with 0 (T1), 300 (T2) and 600 mM (T3) NaCl solutions. The values represent means ± SE (*n* = 3 for control treatments, germination without salt; *n* = 5 for seed germination in 100 mM NaCl; *n* = 10 for all other treatments). Different lowercase letters indicate significant post hoc pairwise differences according to the Tukey test at *p* ≤ 0.05 between salt treatments in the greenhouse (treatment), seed germination conditions (origin) and their interaction (T × O).

Chlorophyll *a* content increased significantly with salinity, from an average of 1.10 mg g^−1^ DW in controls to 1.58 mg g^−1^ DW at 300 mM NaCl, remaining stable at 600 mM (~40% higher than controls; Figure 3a). Chlorophyll *b* showed a similar pattern, rising from 0.29 mg g^−1^ DW in controls to 0.41 mg g^−1^ DW at 600 mM NaCl (Figure 3b). Carotenoid content also increased significantly under high salinity, from 0.27 mg g^−1^ DW in the control group to 0.38 mg g^−1^ DW at 600 mM. Chlorophylls *a* and *b* levels also showed significant differences according to the origin of plants (Table 4), with average values lower in those originated from seeds germinated in the absence of salt than in plants grown from salt-treated seeds (Figure 3a,b). In contrast, carotenoid concentrations did not vary significantly depending on the different germination conditions (Figure 3c; Table 4).

**Figure 4:**
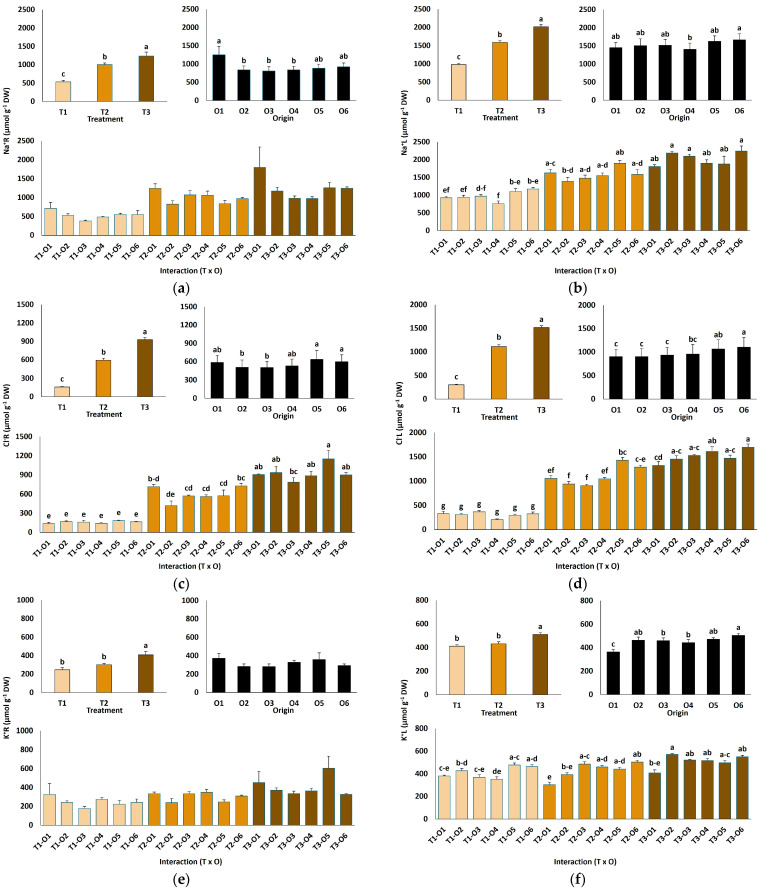
Ion content in roots (**a**,**c**,**e**) and leaves (**b**,**d**,**f**) of *Limonium irtaense* plants grown from seeds germinated in the indicated conditions (O1: 0 mM NaCl, O2: 50 mM NaCl, O3: 100 mM NaCl, O4: R100, O5: R200, and O6: R300, where R100, R200, and R300 refer to germination recovery treatments) after one month of watering with 0 (T1), 300 (T2) and 600 mM (T3) NaCl solutions. The values represent means ± SE (*n* = 3 for control treatments, germination without salt; *n* = 5 for seed germination in 100 mM NaCl; *n* = 10 for all other treatments). Different lowercase letters indicate significant post hoc pairwise differences according to the Tukey test at *p* ≤ 0.05 between salt treatments in the greenhouse (treatment), seed germination conditions (origin) and their interaction (T × O).

NaCl treatments significantly affected ion accumulation in roots and leaves of *L. irtaense* plants (Figure 4). Root Na^+^ concentrations increased approximately 2.3-fold, rising from an average of 534 µmol g^−1^ DW in control plants to 1239 µmol g^−1^ DW at the highest salt concentration tested (Figure 4a). Similarly, leaf Na^+^ levels approximately doubled, from 979 µmol g^−1^ DW in the controls to 2019 µmol g^−1^ DW at 600 mM NaCl (Figure 4b). Chloride (Cl^−^) concentrations also increased markedly under high salinity, with 5.8-fold and 5-fold increases observed in roots and leaves, respectively, at the highest NaCl concentration (Figure 4c,d). Na^+^ and Cl^−^ concentrations were significantly higher in leaves than in roots, indicating the presence of mechanisms for the active transport of the ions to the aboveground plant tissues (Figure 4a–d).

The origin of plants had a much weaker effect on the patterns of Na^+^ and Cl^−^ accumulation than the salt treatments, both in roots and leaves. The general observed trend was that the plants grown from seeds germinated in water showed higher average concentrations of both ions in roots than those coming from salt-treated seeds, whereas this trend was reversed in leaves; however, in most cases, the differences between the different plant origins were not statistically significant (Figure 4a–d). This pattern suggests that salt-priming of the seeds may slightly enhance the active transport of Na^+^ and Cl^−^ from roots to leaves in the derived young plants.

The applied salt treatments increased root and leaf potassium (K^+^) contents in a concentration-dependent manner, although the differences with the non-treated controls were statistically significant only in the presence of 600 mM NaCl (Figure 4e,f). Notably, plants originating from salt-germinated seeds or the recovery of germination tests exhibited a superior capacity to maintain elevated foliar K^+^ levels, with significant differences with the controls observed at 300 and 600 mM NaCl (Figure 4f).

**Figure 5:**
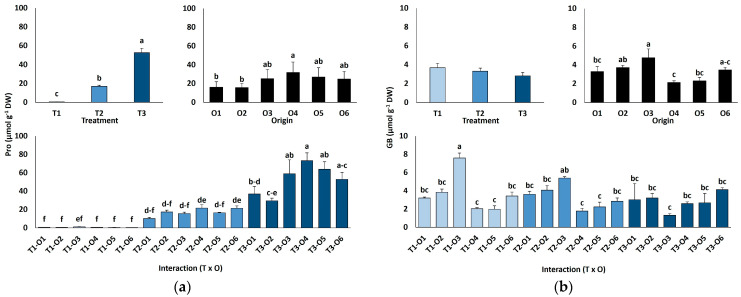
Proline (**a**) and glycine betaine (**b**) contents of *Limonium irtaense* plants grown from seeds germinated in the indicated conditions (O1: 0 mM NaCl, O2: 50 mM NaCl, O3: 100 mM NaCl, O4: R100, O5: R200, and O6: R300, where R100, R200, and R300 refer to germination recovery treatments) after one month of watering with 0 (T1), 300 (T2) and 600 mM (T3) NaCl solutions. The values represent means ± SE (*n* = 3 for control treatments, germination without salt; *n* = 5 for seed germination in 100 mM NaCl; *n* = 10 for all other treatments). Different lowercase letters indicate significant post hoc pairwise differences according to the Tukey test at *p* ≤ 0.05 between salt treatments in the greenhouse (treatment), seed germination conditions (origin) and their interaction (T × O).

Leaf proline (Pro) contents increased sharply with salinity, from an average value of 0.66 µmol g^−1^ DW in the controls to 17.1 µmol g^−1^ DW at 300 mM NaCl, peaking at 52.6 µmol g^−1^ DW in the presence of 600 mM NaCl (80-fold increase; Figure 5a). There was also a significant variation depending on plant origin, with the highest Pro value calculated for plants grown from seeds subjected to the recovery treatment after exposure to 100 mM NaCl (O4). When considering all combinations of salt treatments and plant origin, the differences in Pro accumulation associated with seed germination conditions were most pronounced in plants treated with 600 mM NaCl (Figure 5a).

Glycine betaine (GB) contents were very low under all tested experimental conditions, with average values below 4 µmol g^−1^ DW and showing no significant differences between the salt treatments (Figure 5b). Seed germination conditions and their interaction with the salt treatments had significant effects on GB concentrations (Table 4), but without clear variation patterns (Figure 5b).

Total soluble sugars (TSS) and malondialdehyde (MDA) levels showed no significant changes in response to the salt treatments, regardless of the different plant origins or when considering all combinations of both factors, as indicated by the ANOVA results (Table 4).

**Figure 6:**
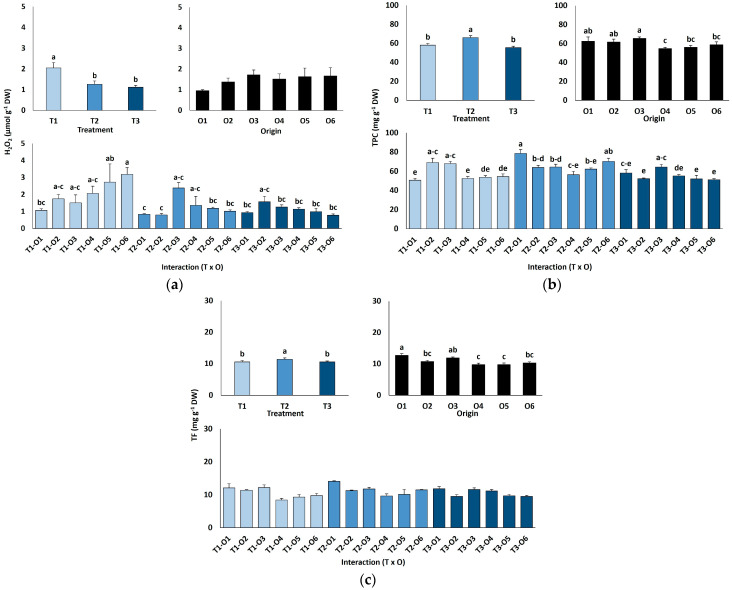
Hydrogen peroxide content (**a**), total phenolic compounds (**b**) and total flavonoids content (**c**) of *Limonium irtaense* plants grown from seeds germinated in the indicated conditions (O1: 0 mM NaCl, O2: 50 mM NaCl, O3: 100 mM NaCl, O4: R100, O5: R200, and O6: R300, where R100, R200, and R300 refer to germination recovery treatments) after one month of watering with 0 (T1), 300 (T2) and 600 mM (T3) NaCl solutions. The values represent means ± SE (*n* = 3 for control treatments, germination without salt; *n* = 5 for seed germination in 100 mM NaCl; *n* = 10 for all other treatments). Different lowercase letters indicate significant post hoc pairwise differences according to the Tukey test at *p* ≤ 0.05 between salt treatments in the greenhouse (treatment), seed germination conditions (origin) and their interaction (T × O).

Leaf hydrogen peroxide (H_2_O_2_) contents were low and did not differ by plant origin but declined significantly with salinity, from 2.1 µmol g^−1^ DW in controls to 1.1 µmol g^−1^ DW at 600 mM NaCl (Figure 6a).

Leaf contents of antioxidant metabolites were also determined (Figure 6b,c). Total phenolic compounds (TPC) concentrations rose slightly but significantly at 300 mM NaCl (66 mg eq. GA g^−1^ DW) but returned to control levels (56–58 mg eq. GA g^−1^ DW) in the presence of 600 mM NaCl (Figure 6b). The effects of seed germination conditions and the interactions between plant origin and salt treatments were small, although statistically significant according to the ANOVA results (Table 4); however, no clear patterns of variation were observed (Figure 6b). Changes in total flavonoids (TF) content in response to salt treatments or different plant origins followed similar patterns to those of TPC, although the interaction of the two factors was not significant (Table 4, Figure 6c).

### 2.5. Principal Component Analysis

**Figure 7:**
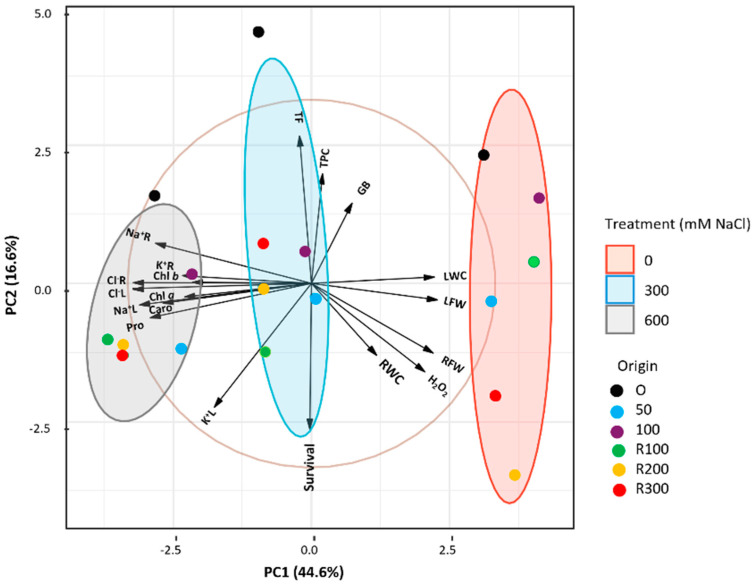
Principal Component Analysis biplot of growth and biochemical data of *Limonium irtaense*. Samples are grouped according to the applied NaCl treatments: 0, 300, and 600 mM, represented by red, blue, and grey 95% confidence ellipses, respectively. Origin: plants derived from seeds germinated without salt (black circles) or in the presence of 50 (blue circles) or 100 (purple circles) mM NaCl; R100, R200, and R300 refer to germination recovery treatments, i.e., plants derived from seeds that did not germinate in the presence of 100, 200 or 300 mM NaCl and were subsequently germinated in distilled water before transplanting. Abbreviations: root fresh weight (RFW), leaf fresh weight (LFW), root water content (RWC), leaf water content (LWC), chlorophylls *a* and *b* (Chl *a* and Chl *b*), carotenoids (Caro), proline (Pro), glycine betaine (GB), hydrogen peroxide (H_2_O_2_), total phenolic compounds (TPC), total flavonoids (TF), sodium in roots (Na^+^R), sodium in leaves (Na^+^L), chloride in roots (Cl^−^R), chloride in leaves (Cl^−^L), potassium in roots (K^+^R), and potassium in leaves (K^+^L).

A Principal Component Analysis (PCA) was conducted on the most significant morphological and biochemical parameters (Figure 7) to assess the combined effects of salt stress and plant origin. Five components with eigenvalues greater than 1 explained 87.97% of the total variation; PC1 and PC2 accounted for 44.6% and 16.6%, respectively. The biplot showed a clear separation of the treatments (0, 300, 600 mM NaCl): controls clustered on the right, whereas both salt treatments grouped to the left, reflecting treatment-dependent shifts in physiological and biochemical traits. Ellipses highlighted consistent clustering patterns. Leaf water content (LWC) and leaf fresh weight (LFW) had strong positive loadings on PC1, separating the control group, whereas Na^+^ and Cl^−^ levels in roots and leaves, photosynthetic pigments, and proline (Pro) had negative loadings, aligning with salt-stressed plants.

Seedling survival rate, total phenolic content (TPC), and total flavonoid content (TF) contributed more to PC2, distinguishing variation along this axis. While control and 300 mM NaCl groups showed greater dispersion, plants from non-saline germination consistently appeared on the positive side of PC2, indicating distinct physiological responses compared to those germinated under salinity or recovery conditions.

## 3. Discussion

Knowledge on the ecophysiology and stress responses of rare and threatened species is fundamental for their effective conservation [26]. For species such as *L. irtaense*, long-term survival depends on preserving natural habitats and selecting suitable sites for translocation or population reinforcement. A species’ ability to establish and persist in a new location is directly influenced by favourable environmental conditions that support seed germination, vegetative growth, and reproductive success [27]. Therefore, conservation strategies must incorporate both biotic and abiotic factors, including habitat structure, reproductive biology, population dynamics, as well as the evaluation of the plants’ tolerance to stress conditions [28,29]. Determination of the physiological and biochemical responses of *L. irtaense* to high salinity is relevant, and not only for the conservation of this highly threatened endemic species, which has not been previously studied. In addition, it belongs to a genus, *Limonium*, showing important qualitative and quantitative differences in the mechanisms of stress tolerance between its taxa, regarding, for example, the use of particular functional osmoprotectants, the presence of specific ion transport mechanisms, or the activation of different enzymatic and non-enzymatic antioxidant defences [30].

### 3.1. Germination Responses to Salt Stress and Halopriming Effects

Germination was highest in the absence of NaCl, declining progressively with increasing salinity. For *L. irtaense*, as in many halophytes, the threshold for a substantial inhibition of germination (<20% germinated seeds) is about 200 mM NaCl [30]. Similar patterns have been reported for *L. caesium* [31], *L. cossonianum* [32], *L. mansanetianum* [33], *L. delicatulum*, *L. supinum* [34], and *L. tabernense* [35]. Some species exhibit enhanced germination at low salinity (50–100 mM NaCl; [36]), while others tolerate higher salt concentrations; for example, *L. stocksii* reaches 60% germination at 300 mM NaCl [37]. Seeds that failed to germinate in the presence of salt remained viable and germinated in distilled water, indicating salt-induced dormancy, an adaptation that allows germination to be delayed until soil conditions improve. This strategy, common in coastal halophytes, maintains seed banks capable of regenerating when rainfall reduces soil salinity [38,39].

Mean germination time (MGT) was shorter in seeds exposed to salt and then transferred to distilled water than in seeds germinated directly in water or in the presence of NaCl. This behaviour resembles halopriming, where a mild salt pre-treatment improves tolerance and accelerates germination [40]. Although recovery occurs post-stress rather than pre-sowing, both processes seem to “condition” seeds through persistent stress-related metabolic changes, enhancing germination speed and uniformity, and improving establishment in fluctuating saline environments [41,42].

### 3.2. Stress Memory and Seedling Survival

One of the most striking outcomes of this study was the significantly higher survival of seedlings that were either germinated in the presence of moderate salt concentrations (50–100 mM NaCl) or underwent recovery of germination after salt exposure. This result suggests that *L. irtaense* can develop a form of stress memory, where exposure to stress during early stages of development enhances tolerance during subsequent growth. Stress memory in plants is often mediated through hormonal and molecular mechanisms [43,44]. Even a short period of stress during seed germination can initiate lasting physiological and biochemical adjustments that improve post-transplantation performance [45].

### 3.3. Plant Growth in the Presence of Different NaCl Concentrations

After germination, plants generally exhibit higher salt tolerance as they develop [46], which is best assessed by the impact of salinity on vegetative growth. Growth reduction under stress is a common adaptive strategy, reallocating resources from biomass accumulation to defence mechanisms [47]. In our study, *L. irtaense*’s growth declined, slightly but significantly, under salt treatments, with maximum growth observed in control plants irrigated with tap water, a pattern similar to those reported for *L. dufourii* [48] and *L. angustebracteatum* [49]. However, moderate salinity can enhance biomass in some species, such as *L. albuferae* [29] and *L. pectinatum* [50]. Certain taxa, including *L. stocksii*, maintain growth up to 300 mM NaCl [51], whereas *L. virgatum* showed a 150% growth increase at 400 mM NaCl and stable biomass even at 800 mM [36].

Coming back to *L. irtaense*, although the effect on growth of plant origin (that is, the previous seed germination conditions) was weaker than that of the salt treatments, it was nonetheless significant. Average root and leaf fresh weights were lower in seedlings generated from control seeds germinated directly in water than in those from seeds germinated in the presence of moderate salt concentrations or from the germination recovery assays, indicating that the seedlings derived from salt-primed seeds not only showed significantly higher survival rates after transplanting to pots but also appear to be slightly more tolerant to salt.

### 3.4. Biochemical Responses to Salt Stress

The physiological and biochemical responses of *L. irtaense* to salinity observed in our experiments, discussed below, are consistent with well-established mechanisms of salt tolerance in halophytes. They include counteracting ion toxicity by a tight control of ion uptake, transport, and compartmentalisation, sequestering excess Na^+^ and Cl^−^ in vacuoles to protect the cytosol, while maintaining a high K^+^/Na^+^ ratio to support normal metabolic activity under salt stress conditions [19]. In addition, osmolytes such as proline play important roles in abiotic stress defence through osmotic adjustment and acting as osmoprotectans, ROS scavengers and signalling molecules [24,25]. Furthermore, high salinity, as other abiotic stresses, generally generate oxidative stress as a secondary effect, inducing the activation of antioxidant systems in the stressed plants; however, highly tolerant species, such as many *Limonium* taxa, may possess efficient mechanisms to cope with elevated salinity preventing the accumulation of too high ROS levels, therefore not requiring the activation of strong antioxidant defences [30], which seems to be also the case for *L. irtaense*.

### 3.5. Ion Transport and Homeostasis

*Limonium* species use multiple salt tolerance strategies, including control of ion transport. They reduce toxic ions contents in photosynthetic tissues via specialised salt-excreting glands [30,52] and often act as salt accumulators, storing Na^+^ and Cl^−^ in vacuoles of aboveground tissues [53,54,55]. In glycophytes, Na^+^ above 100 mM inhibits protein synthesis and other cellular processes, whereas vacuoles in tolerant plants can hold 0.5–2 M Na^+^, compartmentalising excess ions through specialised transporters [56,57,58]. Halophytes absorb and retain essential nutrients even under high external Na^+^ and Cl^−^, using these ions not only for tolerance but also for osmotic regulation, driving substantial ion fluxes [20,59].

A high cytoplasmic K^+^/Na^+^ ratio is critical under salinity because Na^+^ competes with K^+^ at protein-binding sites and affects ion channel function [60,61]. Salt stress can also trigger programmed cell death [62]. Plants reduce root Na^+^ toxicity by moving excess Na^+^ to shoots, where it functions as an inexpensive osmoticum [58,63]. Although Na^+^ often competes with K^+^ uptake such that cellular K^+^ concentrations generally decrease with increasing salinity, several *Limonium* species actively transport K^+^ to leaves under high salinity conditions, maintaining or even increasing leaf K^+^ levels [36,48]. Our results show significant K^+^ increases in both roots and leaves of *L. irtaense* under salt stress, confirming K^+^ retention as a key mechanism of tolerance. The only origin-related difference observed was the higher leaf K^+^ concentrations measured in plants from seeds germinated directly in salt or from the recovery of germination assays, which provides additional support to the notion that early salt exposure primes plants for improved tolerance, at least partly through a more efficient mechanism of K^+^ retention at high salinity.

### 3.6. Osmolyte Accumulation

Osmolyte accumulation is vital for salinity tolerance, contributing not only to osmotic balance but also to additional tolerance mechanisms such as ROS scavenging or cellular signalling [20,25]. Proline (Pro) is one of the most common osmolytes, aiding in osmotic regulation, protein and membrane stability, ROS detoxification, and stress signalling [64,65]. In *L. irtaense*, Pro levels rose under salt stress, with higher accumulation in plants obtained from seeds germinated under, or recovered from, saline conditions. While Pro accumulation is widespread in *Limonium* under salt or drought stress, its physiological role is debated: in *L. latifolium*, it may be linked more to damage repair than osmoprotection [66]. A comparative study of two endemic *Limonium* species from the Valencian region found that the more stress-sensitive species accumulated higher Pro levels under all tested conditions [55]. Conversely, a different study focusing on four other *Limonium* species from the same area found that the species exhibiting the highest salt tolerance accumulated greater Pro concentrations when exposed to salt stress [36]. The increased Pro contents in *L. irtaense* plants grown from salt-germinated seeds or recovery treatments may also contribute to their relatively higher salt tolerance as compared to those derived from seeds germinated directly in water.

Glycine betaine (GB), another key osmoprotectant abundant in Amaranthaceae, has multiple stress-related roles [67]. In our study, GB levels did not vary with salt treatments but differed by plant origin without a clear trend. Overall GB concentrations in *L. irtaense* were notably lower than in other *Limonium* species under similar stress conditions [36], in fact, too low to have any significant osmotic effect, suggesting GB is not a major contributor to *L. irtaense* salt tolerance.

### 3.7. Reactive Oxygen Species and Antioxidant Responses

Abiotic stresses such as high salinity increase reactive oxygen species (ROS) production, which can serve as signalling molecules but cause cellular damage when excessive [68]. High ROS levels can damage nucleic acids, proteins, and lipids, with lipid peroxidation, commonly assessed via malondialdehyde (MDA) content, compromising membrane integrity [69]. In our study, MDA levels did not differ significantly between salt treatments or plant origins. Hydrogen peroxide (H_2_O_2_) concentrations were also unaffected by salinity and, on average, were higher in the controls; no origin-related differences were detected. Although MDA’s reliability as a sole oxidative damage marker is debated [70], these results suggest that salt stress did not induce substantial oxidative stress in *L. irtaense*, reducing the need for the activation of antioxidant defence systems.

Under stress, plants synthesise antioxidants such as ascorbic acid, tocopherols, glutathione, carotenoids, and phenolics, including flavonoids, which aid ROS detoxification [71]. In many species, phenolic and flavonoid levels rise under stress [69,72], but some *Limonium* taxa exhibit minimal changes, possibly due to constitutive or alternative ROS control mechanisms [48,73]. This may reflect the inherent capacity of highly salt-tolerant species to limit ROS production, for example, by restricting cytosolic Na^+^ [74]. Similarly, *L. irtaense* showed only small, generally non-significant changes in total phenolic content (TPC) and total flavonoids (TF) in the presence of elevated salt concentrations.

## 4. Materials and Methods

### 4.1. The Species Under Study

*Limonium irtaense* is a perennial plant that grows 40–70 cm tall with a thick, woody caudex and 1–3 stems. The leaves are basal, green at anthesis, and ovate-spathulate to orbicular-elliptic in shape, with visible lateral nerves and mucronate at the apex. The inflorescence is 10–30 cm long, with loose spikes that are 8–20 mm long, and flowers with violet, emarginated petals. The fruit is small, measuring 1.5 × 0.5 mm. Flowering occurs from June to September, while fruiting takes place between July and October [6].

### 4.2. Seed Origin and Limonium Irtaense Populations

The seeds used in this study were provided by the Centre of Forestry Research and Experimentation (CIEF, Regional Government of Valencia, Spain), from accession C401-C114A. The seeds were collected from the only natural population of the species, located in Serra de Irta, near the city of Peñíscola, Castellón province, at geographic coordinates 4472373/788133, on 21 September 2012 (Supplementary Figure S1, Core 1).

Serra de Irta is a mountainous alignment parallel to the coastline, part of the Catalan coastal mountain range, with an NNE–SSW orientation. It has an average width of 7 km and a length of approximately 20 km. The relief of the mountain range consists mainly of Jurassic and Cretaceous carbonate materials and Quaternary detrital accumulations of small magnitude.

The same seeds had been used by CIEF to successfully establish five translocated populations of the species in Serra de Irta (Figure S1, 1–5), although translocation efficiency needs to be improved. The new locations differ in soil properties such as texture, electrical conductivity, organic matter content or cation exchange capacity (Supplementary Materials, Table S1).

### 4.3. Soil Analysis

From each location (Core 1, and translocated populations 1 to 5), three soil samples were collected at 0–10 cm depth in the root zones of *Limonium* plants. Samples were weighed, air-dried for one week, and then sieved through a 2 mm mesh. A subsample was oven-dried at 105 °C for 24 h to calculate moisture content as the ratio of air-dry to oven-dry weight. Coarse element percentage was determined by weighing material retained on the sieve. Soil texture was analysed using the hydrometer method [75]. Organic matter (OM) was measured using the Walkley and Black wet oxidation method [76], and carbonate content was determined with a Bernard calcimeter. Soil pH (1:2.5 *w*/*v*) was determined with a Crison Basic 20 pH meter, and electrical conductivity (EC) in a 1:5 *w*/*v* aqueous extract with a Crison Basic 30 conductivity meter (Crison Instruments, Alella, Spain). Cation exchange capacity (CEC) followed Rhoades [77], involving saturation with sodium-buffered solution (pH = 8.2), extraction with MgNO_3_, and subsequent Na^+^ and Cl^−^ analyses.

### 4.4. Seed Germination and Recovery of Germination

For the germination trials, seeds were placed in standard 90 mm Petri dishes containing two layers of filter paper. The papers were moistened with either 5 mL of distilled water (serving as the control) or with solutions of 50, 100, 200, and 300 mM NaCl, to impose increasing levels of salt stress. Each experimental condition included four replicates, with 25 seeds placed in each Petri dish. Germination was carried out in a controlled environment using an EGH1501HR germination chamber (EQUiTEC, Madrid, Spain), with a temperature cycle set to 30 °C for 16 h and 20 °C for 8 h, maintaining a relative humidity of 65%.

Germination was monitored daily for 15 days. A seed was considered germinated once its radicle reached a length of at least 2 mm. Germination capacity was calculated as the percentage of seeds that successfully germinated. To assess the germination rate, the mean germination time (MGT) was determined using the method described by Ellis and Roberts [78], calculated by the formula:

MGT = ∑Dn/∑n(1)

where D is the number of days from the beginning of the experiment, and n is the count of seeds that germinated on day D.

Seeds that failed to germinate in the presence of 100, 200 or 300 mM NaCl were subjected to recovery assays. These seeds were thoroughly rinsed to eliminate salt residues, then transferred to fresh Petri dishes moistened with distilled water and incubated under the same conditions as the original test for another 15 days.

### 4.5. Plant Growth in the Greenhouse

Seedlings from the initial germination tests and the recovery of germination assays were used to evaluate plant growth. These seedlings were carefully transplanted from the Petri dishes into individual pots (5.5 × 6 cm), each labelled with the treatment applied during the germination phase and the treatment to be applied at this growth stage. The pot substrate consisted of a mixture of peat and vermiculite in a 3:1 ratio.

A two-month acclimatisation period was allowed between transplanting and the initiation of salt treatments, ensuring that the seedlings reached a sufficiently developed size. During this time, the survival of seedlings was recorded to evaluate their resilience following transplantation.

The salt treatments were applied for one month through three weekly irrigations. Each plant group, derived from the initial germination tests and the germination recovery assays, was watered with one of the following irrigation solutions: tap water (control) or 300 and 600 mM NaCl solutions (for the salt treatments). The number of replicates in each group varied according to the number of plants available after transplanting since the survival rate differed depending on the previous germination conditions (see Table 2). Thus, *n* = 3 per treatment for plants derived from seeds germinated in the absence of salt; *n* = 5 for plants derived from seeds germinated at 100 mM NaCl; and *n* = 10 for those from seeds germinated in the presence of 50 mM NaCl and from the germination recovery assays, which showed a higher survival rate after transplantation into pots. All plants were harvested for further analysis one month after the start of the treatments.

To evaluate the effects of salt stress on plant development, several growth-related parameters were recorded, including the fresh weight of the roots and leaves. To calculate the plant water content, a portion of root and leaf tissues was oven-dried at 65 °C until a stable weight was achieved. The water content was then determined by calculating the ratio of fresh to dry weight.

### 4.6. Biochemical Analyses

Photosynthetic pigments were extracted from approximately 0.1 g of fresh leaf tissue (previously ground) using 1 mL of ice-cold 80% (*v*/*v*) acetone, following the procedure established by Lichtenthaler and Wellburn [79]. Pigment concentrations were determined and expressed in milligrams per gram of dry weight (mg g^−1^ DW).

The levels of monovalent ions (Na^+^, K^+^, Cl^−^) were analysed in both root and leaf tissues, following the methodologies of Weimberg [80] and Cotlove [81]. For this, 0.1 g of oven-dried, ground material was extracted in boiling Milli-Q water, cooled on ice, and centrifuged for 10 min at 13,300× *g* at room temperature. Cation concentrations were measured using a Corning 410 Classic flame photometer (Corning Inc., New York, NY, USA), whereas chloride levels were quantified with a Sherwood 926 chloride analyser (Sherwood Scientific Ltd., Cambridge, UK).

Proline (Pro) accumulation was quantified using the protocol of Bates et al. [82], in which 0.1 g of fresh, ground leaf tissue was extracted with 0.5 mL of a 3% (*w*/*v*) sulphosalicylic acid solution. Results were expressed in micromoles per gram of dry weight (µmol g^−1^ DW).

Glycine betaine (GB) content was determined based on the method proposed by Grieve and Grattan [83], with slight modifications [84].

Quantification of total soluble sugars (TSS), malondialdehyde (MDA), total flavonoids (TF), and total phenolic compounds (TPC) was carried out using 80% leaf methanolic extracts.

TSS content was determined using the colourimetric method of Dubois et al. [85], with concentrations calculated from a glucose standard curve and expressed as mg equivalent of glucose per gram of dry weight (mg eq. glucose g^−1^ DW).

Malondialdehyde (MDA) content was determined based on the procedure outlined by Hodges et al. [86]. Methanolic extracts were reacted with 0.5% thiobarbituric acid (TBA) prepared in 20% trichloroacetic acid (TCA), and MDA levels were calculated using the equation provided by Taulavuori et al. [87].

Hydrogen peroxide (H_2_O_2_) levels were quantified following the method of Loreto and Velikova [88]. Fresh leaf tissues were homogenised in 0.1% (*w*/*v*) trichloroacetic acid, and the supernatant obtained was combined with a 10 mM potassium phosphate buffer (pH 7.0) and 1 M potassium iodide (KI). Hydrogen peroxide (H_2_O_2_) levels were quantified using a standard calibration curve and reported as micromoles per gram of dry weight (µmol g^−1^ DW).

Total phenolic compounds (TPC) content was determined using the Folin–Ciocalteu reagent in the presence of sodium carbonate (Na_2_CO_3_), according to the method outlined by Blainski et al. [89], and expressed in terms of equivalents of gallic acid (GA), used as the standard (mg eq. GA g^−1^ DW). Total flavonoid (TF) content was assessed using the method of Zhishen et al. [90], which involves the nitration of catechol-containing compounds with sodium nitrite (NaNO_2_), followed by complexation with aluminium chloride (AlCl_3_) under alkaline conditions. Flavonoid levels were quantified against a catechin calibration curve and expressed as catechin equivalents (mg eq. C g^−1^ DW).

### 4.7. Statistical Analysis

Statistical analyses were conducted using Statgraphics Centurion XVIII (Statpoint Technologies, Warrenton, VA, USA). Data normality was assessed using the Shapiro–Wilk test, and homogeneity of variances was evaluated with Levene’s test. Germination percentages were arcsine square root transformed, and effects of salt treatments on germination rate and mean germination time (MGT) were analysed via one-way ANOVA; significant differences were examined with Tukey’s post hoc test (*p* ≤ 0.05). For growth and biochemical parameters not meeting normality or homoscedasticity assumptions, a logarithmic transformation was applied before ANOVA. A two-way ANOVA was used to evaluate the effects of treatment, plant origin (i.e., seed germination conditions), and their interaction, followed by Tukey’s test for multiple comparisons (*p* ≤ 0.05). Principal Component Analysis (PCA) of morphological and biochemical variables with significant effects was performed and visualised using SRplot [91].

## 5. Conclusions

Taken together, our results suggest that *L. irtaense* salt tolerance is mainly based on the transport of toxic ions to the shoots and sequestration in leaf vacuoles, K^+^ retention, and high Pro accumulation in photosynthetic tissues. Even though deeper molecular studies, including analysis of the expression of specific genes involved in these processes (e.g., encoding ion transporters) will be required to further elucidate regulatory networks, the present physiological and biochemical evidence is consistent with established halophyte adaptation strategies and supports our interpretations of salt-priming and stress tolerance mechanisms in this endangered species.

In situ conservation of the highly threatened *L. irtaense* requires the establishment of new populations via translocation, introducing ex situ produced plants or sowing seeds into suitable selected sites where a relatively large number of plants could survive the challenging early phases of seed germination and seedling establishment.

Our results have important implications for *L. irtaense* and other halophytes conservation programmes. Early exposure to moderate salinity, in natural habitats or under controlled nursery conditions, can improve seedling post-translocation survival and enhance the plants’ salt stress tolerance, as shown by the present study. A deeper knowledge of the plants’ responses to stress should enable better site selection and management. The observed effects of seed exposure to moderate salt concentrations and the germination recovery tests support the incorporation of priming-like methods into conservation protocols. Conditioning seeds or seedlings before planting may improve establishment success in degraded or marginal coastal habitats, where salinity, nutrient limitations, and fragmentation remain as significant challenges.

## Figures and Tables

**Table 1:** Final germination percentages and mean germination time (MGT) of *Limonium irtaense* seeds in germination and recovery assays. Different letters denote statistically significant differences between means according to the Tukey test. Values shown are means per plate ± SE; *n* = 4. Recovery treatments (R100, R200, R300) refer to seeds that did not germinate in the presence of 100, 200 or 300 mM NaCl, respectively, and were then washed with distilled water before the germination test in water.

Treatment (mM NaCl)	Germination (%)	MGT (Days)
0	80.00 ± 1.63 c	3.50 ± 0.21 e
50	53.00 ± 8.22 d	6.66 ± 0.32 bc
100	35.00 ± 3.41 e	7.04 ± 0.81 b
200	19.00 ± 2.51 f	5.39 ± 0.32 d
300	7.00 ± 1.91 g	9.04 ± 0.82 a
R100	93.75 ± 0.00 a	2.36 ± 0.02 e
R200	98.50 ± 0.00 a	2.51 ± 0.05 e
R300	89.06 ± 4.50 ab	2.36 ± 0.15 e

**Table 2:** Survival of seedlings from different germination treatments one month after transplanting into pots in the greenhouse. Values represent the number of seedlings initially transplanted and the percentage that survived after one month. R100, R200, and R300 refer to seedlings from the recovery treatments, i.e., seeds that did not germinate in the presence of 100, 200 and 300 mM NaCl, respectively, and were then washed and germinated in water.

Treatment (mM NaCl)	Number of Transplanted Seedlings	Number of Seedlings Surviving After One Month	Survival Percentage
0	80	9	11.3%
50	53	32	60.4%
100	35	17	48.6%
R100	45	35	77.8%
R200	67	49	73.1%
R300	56	41	73.2%

**Table 3:** Two-way analysis of variance (ANOVA) of treatment, origin, and their interactions for the growth parameters analysed. Significant *p*-values (*p* ≤ 0.05) are shown in bold. Abbreviations: RFW, root fresh weight; LFW, leaf fresh weight; RWC, root water content; LWC, leaf water content.

Trait	Treatment		Origin		Treatment × Origin	
	F	*p*-Value	F	*p*-Value	F	*p*-Value
RFW	15.02	**0.000**	4.98	**0.000**	3.07	**0.001**
LFW	12.31	**0.000**	3.84	**0.002**	0.84	0.591
RWC	1.56	0.213	0.97	0.436	2.80	**0.003**
LWC	17.01	**0.000**	2.28	**0.050**	2.34	**0.014**

**Table 4:** Two-way analysis of variance (ANOVA) of treatment, origin, and their interactions for the biochemical parameters analysed. Significant *p*-values (*p* ≤ 0.05) are shown in bold. Abbreviations: Chl *a*, chlorophyll *a*; Chl *b*, chlorophyll *b*; Caro, carotenoids; Na^+^R, sodium in roots; Na^+^L, sodium in leaves; Cl^−^R, chloride in roots; Cl^−^L, chloride in leaves; K^+^R, potassium in roots; K^+^L, potassium in leaves; Pro, proline; TSS, total soluble sugars; GB, glycine betaine; MDA, malondialdehyde; H_2_O_2,_ hydrogen peroxide; TPC, total phenolic compounds; TF, total flavonoids.

Trait	Treatment (df = 2)		Origin (df = 5)		Treatment × Origin (df = 17)	
	F	*p*-Value	F	*p*-Value	F	*p*-Value
Chl *a*	70.49	**0.000**	3.14	**0.019**	6.27	**0.000**
Chl *b*	64.06	**0.000**	2.66	**0.038**	4.64	**0.000**
Caro	40.99	**0.000**	0.97	0.448	3.54	**0.002**
Na^+^R	33.25	**0.000**	3.55	**0.010**	1.01	0.457
Na^+^L	178.66	**0.000**	3.35	**0.014**	2.93	**0.009**
Cl^−^R	281.25	**0.000**	1.27	**0.034**	3.03	**0.007**
Cl^−^L	870.18	**0.000**	8.60	**0.000**	7.55	**0.000**
K^+^R	13.54	**0.000**	1.53	0.204	1.89	0.080
K^+^L	54.51	**0.000**	22.36	**0.000**	7.47	**0.000**
Pro	143.50	**0.000**	4.12	**0.005**	2.91	**0.009**
TSS	0.23	0.798	2.47	0.051	2.04	0.057
GB	3.18	0.054	8.16	**0.000**	5.85	**0.000**
MDA	2.86	0.07	2.46	0.051	1.80	0.090
H_2_O_2_	11.63	**0.000**	1.88	0.122	3.06	**0.007**
TPC	23.63	**0.000**	6.80	**0.000**	6.55	**0.000**
TF	1.62	**0.046**	9.89	**0.000**	2.02	0.060

## Data Availability

The datasets generated during and/or analysed during the current study are available in the Mendeley repository, https://doi.org/10.17632/gnb6hh34pc.1 (accessed on 21 January 2026).

## Supplementary Materials

The following supporting information can be downloaded at https://www.mdpi.com/article/10.3390/plants15030451/s1, Table S1. Soil parameters analysed in the area of the five *Limonium irtaense* translocated populations (Pop. 1–Pop. 5) and in the natural population (Core 1) in Sierra de Irta (a composite sample of each zone was used for granulometry and cation exchange capacity; n = 3 for the rest of the soil properties). EC_1:5_: electric conductivity (1:5; soil:water); OM: organic matter; CEC: cation exchange capacity. Figure S1. Location of the analysed *Limonium irtense* populations. CORE 1, original natural population. 1 to 5, translocated populations established with seeds from CORE 1.
