# Supplementary material for: Physiological and Biochemical Mechanisms Behind Enhanced Salinity Tolerance in Limonium irtaense Seedlings Following Recovery from Salt Stress
Source: Plants (Basel). 2026 Feb 1;15(3):451. doi: 10.3390/plants15030451 (PMC12898991; doi:10.3390/plants15030451)
Supplement: Supplementary file 1 [file plants-15-00451-s001.zip › plants-4087912-supplementary.pdf]

Table S1. Soil parameters analysed in the area of the five *Limonium irtaense* translocated populations (Pop. 1-Pop. 5) and in the natural population (Core 1) in Sierra de Irta (a composite sample of each zone was used for granulometry and cation exchange capacity; n=3 for the rest of the soil properties). $EC_{1:5}$: electric conductivity (1:5; soil:water); OM: organic matter; CEC: cation exchange capacity

**Parameter**	**Pop. 1**	**Pop. 2**	**Pop. 3**	**Pop. 4**	**Pop. 5**	**Core 1**
Texture class	Sandy loam	Loamy	Sandy loam	Loamy	Clay loam	Sandy loam
Sand (%)	83.3	35.2	67.5	37.4	34	72.6
Silt (%)	12.2	42.3	22.4	39.3	36.8	17.4
Clay (%)	4.5	22.5	10.1	23.3	29.2	10.0
Stoniness (%)	45 ± 5	47 ± 13	45 ± 5	36 ± 12	38 ± 29	39 ± 9
$CaCO_3$ (%)	60 ± 8	52 ± 16	60 ± 8	37 ± 26	35 ± 15	67 ± 7
pH	8.77 ± 0.20	8.47 ± 0.13	8.77 ± 0.20	8.31 ± 0.21	8.33 ± 0.03	8.61 ± 0.07
$EC_{1:5}$ (dS m^{-1})	3.25 ± 1.15	1.72 ± 0.85	3.25 ± 1.15	1.42 ± 0.18	10.13 ± 13.33	2.65 ± 2.57
OM (g kg^{-1})	16.5 ± 7.2	8.2 ± 2.8	16.5 ± 7.2	13.9 ± 4.7	37.8 ± 11.2	11.1 ± 8.9
CEC ($cmol_c$ kg^{-1})	5.91	13.75	9.30	16.12	20.32	9.62

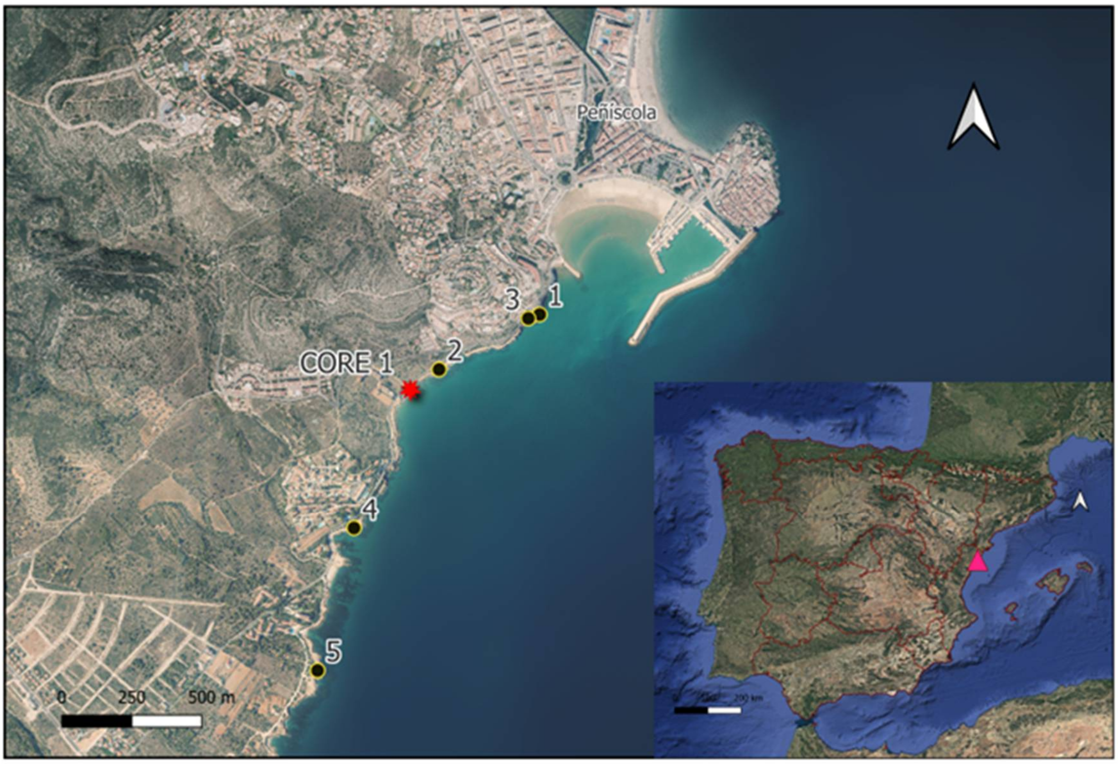

Figure S1. Location of the analysed *Limonium irtense* populations. CORE 1, original natural population. 1 to 5, translocated populations established with seeds from CORE 1
